# The Background of Reduced Face Specificity of N170 in Congenital Prosopagnosia

**DOI:** 10.1371/journal.pone.0101393

**Published:** 2014-07-01

**Authors:** Kornél Németh, Márta Zimmer, Stefan R. Schweinberger, Pál Vakli, Gyula Kovács

**Affiliations:** 1 Department of Cognitive Science, Budapest University of Technology and Economics, Budapest, Hungary; 2 Institute of Psychology, Friedrich-Schiller-University of Jena, Jena, Germany; 3 DFG Research Unit Person Perception, Friedrich Schiller University of Jena, Jena, Germany; Tel Aviv University, Israel

## Abstract

Congenital prosopagnosia is lifelong face-recognition impairment in the absence of evidence for structural brain damage. To study the neural correlates of congenital prosopagnosia, we measured the face-sensitive N170 component of the event-related potential in three members of the same family (father (56 y), son (25 y) and daughter (22 y)) and in age-matched neurotypical participants (young controls: n = 14; 24.5 y±2.1; old controls: n = 6; 57.3 y±5.4). To compare the face sensitivity of N170 in congenital prosopagnosic and neurotypical participants we measured the event-related potentials for faces and phase-scrambled random noise stimuli. In neurotypicals we found significantly larger N170 amplitude for faces compared to noise stimuli, reflecting normal early face processing. The congenital prosopagnosic participants, by contrast, showed reduced face sensitivity of the N170, and this was due to a larger than normal noise-elicited N170, rather than to a smaller face-elicited N170. Interestingly, single-trial analysis revealed that the lack of face sensitivity in congenital prosopagnosia is related to a larger oscillatory power and phase-locking in the theta frequency-band (4–7 Hz, 130–190 ms) as well as to a lower intertrial jitter of the response latency for the noise stimuli. Altogether, these results suggest that congenital prosopagnosia is due to the deficit of early, structural encoding steps of face perception in filtering between face and non-face stimuli.

## Introduction

In prosopagnosia, a neuropsychological condition, the individuals are unable to recognize faces or to make certain decisions about them. Quaglino and Borelli [1] reported the first case of prosopagnosia in 1867, although the term “prosopagnosia” was introduced later by Bodamer [2]. Prosopagnosia has at least two basic different forms. While acquired prosopagnosia (AP) may appear after a stroke, lesion or injury of the occipito-temporal cortex [3]–[11], developmental prosopagnosia (DP) may be present from birth or shortly after that. DP can occur without any brain damage and with normal intelligence or sensory abilities [12]–[19]. In contrast with DP, the congenital form of prosopagnosia (CP) is present from birth [20], and is thought to be due to hereditary impairments. Please note however that to evaluate the onset of the impairment is difficult, if not impossible as of today. The terms DP and CP are often used interchangeably in the literature, and the current study was not designed to discriminate the two forms. Here we adopted the term “congenital” prosopagnosia, merely to signal the fact that the prosopagnosic participants of the present study belong to two generations of the same family, suggesting a role of hereditary factors. Kennerknecht et al. [21] emphasized that CP can be an isolated familial case due to a gene-mutation or a genetically transmitted disorder. However, the distinction of AP, DP and CP is made difficult by the fact that even childhood forms of prosopagnosia can be either hereditary or acquired, attributed to pre- or perinatal episodes such as asphyxia or encephalitis. Therefore it is under heavy debate what cases of prosopagnosia are developmental or congenital and what the differences are between them [21]. A strong argument for the congenital nature of prosopagnosia is that face recognition impairments can be found in more than one members of the same family, stretching across generations. The first case that suggested familiar transmission of CP was reported by McConachie [22]. After that, the next report of a familial history of prosopagnosia was published by De Haan and Campbell [23]. Despite the considerable prevalence of CP in different ethnic populations [21], [24]–[26], the available studies testing prosopagnosia within the same families are very limited as of today.

The visual N170 reflects a negative component of the event-related potential (ERP) peaking about 170 ms after stimulus presentation. The N170 (or its magneto-encephalographic equivalent, the M170 [27], [28]) is the most pronounced over posterior occipito-temporal scalp regions, and marks the earliest difference in amplitude between faces and non-face stimuli. The larger N170 for faces when compared to non-face stimuli is termed as category-sensitivity or “face effect” [29] and this differential signal marks normal early face-categorization processes and as such it is applied widely to test prosopagnosic and neurotypical participants as well [29]–[31], [32], [33]. As of today, very few studies of N/M170 are available in DP/CP participants. A survey of the literature shows that only approximately 50% of the available 30 DP participants showed larger N/M170 for faces than for other stimulus categories such as houses (KL & ML [34] and twelve of the sixteen participants in a recent study [35]), caricatures and houses (LT, NN & TP [36]) or bodies and shoes (GR & HV [37]). For the other half of the DP participants the N/M170 was similar in amplitude for face and non-face stimuli (KW [38]; EB, KNL, NM [34]; ET [36]; JS & CB [37] and 4 of the 16 participants of Towler et al. [35] (but see [19] for a different conclusion showing similar distribution of face selectivity for M170 in neurotypical participants and individuals presenting with CP). Regarding CP participants, no face-selectivity was found at the level of the N170 in any of the previously reported four cases (MZ [39]; YT [40]; SO & GH [13]). Thus, it seems that in DP/CP face recognition impairments are sometimes related to lower face sensitivity of the N170. However, to our best knowledge the available studies of CP within the same families only reported behavioural and neuropsychological results [24], [41]–[43]; unequivocal electrophysiological and functional evidence for the heritability of prosopagnosia has not yet been reported (but see [44]). Here we aimed at evaluating the face-related electrophysiological responses of three members of the same family, a father and his two children, all presenting with CP. In a previous study [44] we have tested the functional properties of their core face processing network [45]–[47] using fMRI and a block-design experiment. We used pictures of faces and artificial objects as stimuli to evaluate whether the face-sensitive mechanisms are preserved in CP. When compared to controls all the three members of the tested family showed reduced face-sensitive BOLD (blood oxygen level-dependent) signal bilaterally in the Fusiform and Occipital Face Areas as well as in the lateral occipital cortex. Furthermore, the dynamic of the fMRI response was also altered when compared to controls in these areas. The specific aim of the current study was to provide further insights into the mechanisms of this reduced face-sensitivity and to reveal the electrophysiological correlates of congenital prosopagnosia. In the present study in addition to faces, we used their Fourier-randomised versions as stimuli (noise), since these stimuli are known to evoke significantly lower N170 amplitudes in neurotypical participants (e.g. [48], [49]). However, a difference in peak ERP amplitude for different stimulus conditions might be due to multiple reasons (see [50], and [31] for a summary). Therefore, to reveal the background of possible impairments of stimulus selectivity, in addition to the conventional ERP peak amplitude and latency measurements, we also tested how the latency of the N170 is affected by stimulus category on the single-trial level.

Finally, recent results showed that the visual ERPs in the 100–200 ms time window might be explained to a large extent by the partial phase resetting of ongoing activity in a restricted frequency band [51]–[54]. It has also been shown that the phase of the cortical oscillations is in connection with the timing of the neural activity in the animal brain [55], however, the exact role of the oscillatory phase and its synchronization in cortical information processing is under heavy debate in the literature. For example, it has been suggested that the synchronization of the EEG shows the timing of the communication between distant neural populations that process the response to the incoming sensory stimuli (for a review see [50]). Accordingly, if the ERPs for face and non-face stimulus categories in CP will not show significant differences, it could be accounted for the increased phase-resetting, or enhanced power after the non-face stimulus. Therefore we also compared the time-frequency properties of the EEG for face and non-face stimulus categories in individuals with CP and neurotypical participants.

We reasoned that if CP is related to an “early filtering deficit” [56], then the prosopagnosic participants will not show differences between face and noise stimuli in the major early face sensitive electrophysiological marker, the N170, that reflects the early, structural encoding stages of face processing [32], [57]. Alternatively, CP may be accounted for by impairments at later processing stages than the visual N170 in which case the earlier electrophysiological markers of CP and neurotypical subjects should be identical. Furthermore, if heritable factors involved in the transmission of CP across generations cause similar underlying neural impairments, then all members of the same family should show similar alterations of the electrophysiological markers. Alternatively, like the previously observed behavioural heterogeneity in prosopagnosia, electrophysiological markers of face processing in CP may also be different, even across family members.

## Materials and Methods

### 2.1. Participants

#### 2.1.1. Congenital prosopagnosic participants

The three CP participants are members of the same family: father (CPf), his son (CPs) and daughter (CPd). CPf is a 56-year-old, right-handed college graduate (education level: 17 years). To the best of his knowledge, he has always had a problem with face recognition, even with his own family members and best friends. CPs is a 25-year-old right-handed male PhD student (actual education level at the time of test: 18 years). By his own admission, he also has always had a problem with recognizing others. CPd is a 22-year-old ambidextrous female undergraduate (actual education level at time of test: 16 years). As far as she could remember, she had face recognition difficulties from early childhood. None of them had any accident, head trauma, or infection of the central nervous system. According to the anecdotal description of the participants, the mother of CPf also reported face recognition difficulties; unfortunately, due to her advanced age (79 years), she could not be tested. All of the participants with CP had normal or corrected-to-normal visual acuity.

#### 2.1.2. Control participants

Due to the age-related differences of the major ERP components that are related to face perception [58]–[61] as well as of the evoked oscillations [62], we recruited 25 age, IQ and education matched neurotypical subjects. The CTRL1 group (n = 18; 1 left handed, 1 ambidextrous, 7 females, mean ± STD age: 24.5±2.1 years) was matched to the two younger CP participants while the CTRL2 group (n = 7, all right handed, 3 females, mean ± STD age: 57.3±5.4 years) was matched to CPf. They gave their informed and written consent to participate in the study, which was approved by the local ethics committee of the Budapest University of Technology and Economics. The subjects' consent was obtained according to the Declaration of Helsinki. None of neurotypical participants had any history of neurological diseases and all had normal or corrected-to-normal visual acuity. Three males and one female from CTRL1 and one male from CTRL2 group were excluded from the further analysis due to the poor quality of EEG recordings. Therefore the current analysis is based on the data of 14 and 6 participants in the CTRL1 and CTRL2 groups, respectively.

### 2.2. Stimulation and Procedure

#### 2.2.1. Neuropsychological assessment

The three CP participants were tested individually with a face perception test battery. First we ruled out general object recognition impairments using the Doors task of the Doors and People test [63]. Furthermore, we evaluated their perceptual performance for age, beauty, gender and similarity with the Philadelphia Face Perception Battery [64] and confirmed the diagnosis of CP by using the Cambridge Face Memory Test (CFMT) [65]; and the Cambridge Famous Faces Test (CFFT) [66]; available online: http://www.faceblind.org/facetests/ff/ff_intro.php. These tests have been validated on DPs (mean CFMT value of 50 non-DP neurotypical subject (± STD)  = 57.90±7.91; [67] and the mean CFFT value (0.89±0.09) from 22 neurotypical participants. Despite severe facial identity recognition impairments, many CP participants recognize facial expressions like neurotypical subjects [40], [41]. To measure a relevant aspect of emotion processing, we applied the Eyes Test [68] in which participants judge 37 subsequently presented eye-regions of different faces and decide which one of four words describes best what the person on the image is thinking or feeling.

#### 2.2.2. EEG experiment

*Stimuli of the EEG experiment*. We used two stimulus categories, faces (FACE) and their Fourier-phase randomized versions (NOISE), each with thirty examples. Faces were full-front view pictures of unfamiliar persons (20–35 years, 16 female), similar to those used in other studies [13], [40], [69]–[71], derived from our database. For NOISE we decreased the phase coherence of these images to 0% [72]. Stimuli subtended a visual angle of 18.65°×18.65° at 72 cm viewing distance and were presented centrally on a uniform gray background.

*Procedure*. After the neuropsychological assessments, participants were tested individually in a dimly lit, sound-attenuated and electrically shielded chamber. Participants performed a face-non-face discrimination task. They had to fixate on the centre of the screen and signal whether the randomly appearing images were FACE or NOISE images by pressing either ‘7’ or ‘8’ button of a standard keyboard with their right hand index or middle fingers (counterbalanced across subjects), respectively. This task was irrelevant regarding the goal of the experiment, and was used to maintain the level of attention constant during the procedure. A trial consisted of a fixation screen (uniform gray oval with a small white cross in the centre; exposition time  = 300 ms), a target (300 ms) and a mask screen (uniform gray picture; 1000–1500 ms). Four blocks were run that consisted of 240 trials overall (2 categories ×30 samples ×4 repetitions). The stimulus presentation was controlled by MATLAB 6.5 (Mathworks, Natick Massachusetts, USA) using the Psychtoolbox 2.54 and custom-made software [73], [74].

### 2.3. Parameters and Data Analysis

#### 2.3.1. EEG recording

The EEG was recorded (Brain Products GmbH., Munich, Germany) over 32 Ag/AgCl electrodes (EasyCap GmbH, Herrsching-Breitbrunn, Germany; 1000 Hz sampling rate; impedance<10 kΩ; reference: AFz) placed according to the extended 10/20 international electrode system [75].

#### 2.3.2. EEG data analysis

The EEG analysis was performed using Brain Vision Analyzer 1.05.0002 (Brain Products GmbH., Munich, Germany) and a custom written Matlab scripts for the single trial analysis and ITC measurements. Eye blink artefacts were corrected mathematically [76] using two EOG channels (Fp1–Fp2). The EEG was re-referenced to average and segmented off-line (1800 ms epochs with 500 ms pre-stimulus baseline). These epochs were visually inspected and all the segments containing ±50µ V voltage drifts were removed. Each segment was baseline-corrected, and filtered using a 24 db/octave high pass filter with a cut-off frequency of 50 Hz. ERPs were averaged for each condition and participant separately and the amplitudes of the N170 was measured as the mean voltage within a 10 ms window, centred on the peak amplitude between 120 and 190 ms after stimulus onset [77]. In order to find the most category sensitive electrode sites we calculated the scalp current density (SCD) that is free of the reference and shows decreased volume conduction, eliminating raw EEG contaminations by saccadic artifacts [78], [79]. To generate SCD waveforms we used Laplacian transforms on the spherical spline-interpolated data. Since previous results usually find the highest N170 amplitude (see [32]) and because the SCD maps of the face-noise differences (FND) showed the maximum unequivocally on P9/10 electrodes for both CTRL1 and CTRL2, the peak latencies of the N170 were extracted at these two occipito-temporal electrodes (P9 (left hemisphere, LH)/P10 (right hemisphere, RH)) at its local minimum. In order not to inflate Type I. error due to the unequal group sizes (14 neurotypical subjects versus 2 CP participants in the young and 6 neurotypical participants versus 1 CP participant in the old neurotypical groups, respectively) the analysis was performed separately for CTRL1 and CTRL2 groups. The amplitude and latency values of each ERP components were submitted to two-way repeated-measures ANOVAs with Stimulus (2; FACE, NOISE) and Hemisphere (2; left and right) as within-subject factors. Post-hoc statistics were performed with Bonferroni tests.

To compare the face specificity directly between CP and CTRL groups, we first determined the difference between the face and noise stimuli (FND) of the N170 by subtracting the amplitude/latency of the N170 component, obtained for noise stimuli (N170_Noise_) from those for face stimuli (N170_Face_) for each participant and electrode separately. The FND values of each CP participant were then compared to the mean FND values of the appropriate neurotypical group using a one-sample t-test and a bootstrap technique with 1000 re-samplings [80]. Bootstrapping can provide a useful tool for analysing single-subject ERPs and it has been shown to prove face-selectivity of the N170 reliably in individual participants [35], [81], [82]. Statistical analysis was performed with SPSS (IBM Corp. Released 2011. IBM SPSS Statistics for Windows, Version 20.0. Armonk, NY: IBM Corp.). For visualization purposes, topographic representations of scalp data and spectrograms were created with the functions of the EEGLAB toolbox for Matlab [83].

#### 2.3.3. Intertrial variance

Estimating the latency distribution of the ERP component across trials allows us to define what properties cause the amplitude differences of the averaged ERPs between conditions [31]. For this analysis, first the latency of the ERP curve was extracted within a window between 120–190 ms [77], using the segmented, artefact rejected and filtered data for each participant individually. Next, we calculated the mean, variance and standard deviations (STD) of N170 latencies across the trials for every participant and stimulus category separately [84]. Finally, the STDs as well as the difference of STD for FACE and NOISE (reflecting its face sensitivity) between neurotypicals and CP participants were compared individually to the means of the neurotypical group, using a one-sample t-test with applying a bootstrap technique with 1000 re-samplings [80]. Intertrial variance analysis was performed using custom written Matlab (The MathWorks Inc., Natick, MA) scripts.

#### 2.3.4. Intertrial Coherence and power analysis

The intertrial coherence (ITC) analysis was performed according to (e.g. [48]). Data analysis was performed using EEGLAB v10.2.5.8b [83] and custom-written Matlab codes. First we segmented (−500 to 1300 ms), baseline corrected, artefact rejected and re-referenced the data to average. Next, we calculated the time-frequency spectrum using a sinusoidal wavelet transform (short-time discrete Fourier transform) computing the power spectrum over the frequency range of 4–40 Hz (interpolated frequency resolution: 0,5 Hz) in a sliding latency window (length of the window: 512 ms, step: 25 ms) and then averaged across trials. We computed the intertrial coherence assessing intertrial phase stability for a given time window and frequency bin as a measure of neural synchrony for each participant and then grand-averaged it for each condition separately. Based on the plotted spectrograms showing ITC changes across the frequency range, average time courses of the theta (4–7 Hz) and alpha (8–12 Hz) frequency bands were estimated. Power was analysed, using the same time and frequency windows as for ITC, as event-related spectral perturbation (ERSP) which detects shifts of the power-spectrum due to the onset of the stimuli [85].

Based on previous results [48], [52], [54], [81] - which explain the enhanced N170 with increased event-related synchronisation in the theta, alpha and beta range within the 0–200 ms time window, we focused our analysis to this time window. Mean ITC and ERSP time courses, corresponding to the N170 ERP component (130–190 ms post-stimulus onset) were submitted to two-way repeated-measures ANOVA over the P9/P10 electrodes with Stimulus (2; FACE, NOISE) and Hemisphere (2; left and right) as within-subject factors. All analyses involved Greenhouse-Geisser adjusted degrees of freedom to correct for violations of the sphericity assumption. Post-hoc analyses were performed using the Bonferroni correction.

To compare the face sensitivity of the ITC and ERSP, we first determined these variables individually in both frequency ranges (theta and alpha) as the difference of mean values between 130 and 190 ms time window after stimulus onset (FND_ITC_: ITC_Face_-ITC_Noise_ and FND_ERSP_: ERSP_Face_-ERSP_Noise_). The individual FND values of the CP participants were then compared to the mean values of the neurotypical groups for all electrodes, using a one-sample t-test with a bootstrap technique with 1000 re-samplings [80].

## Results

### 3.1 Neuropsychological Testing

**Figure 1:**
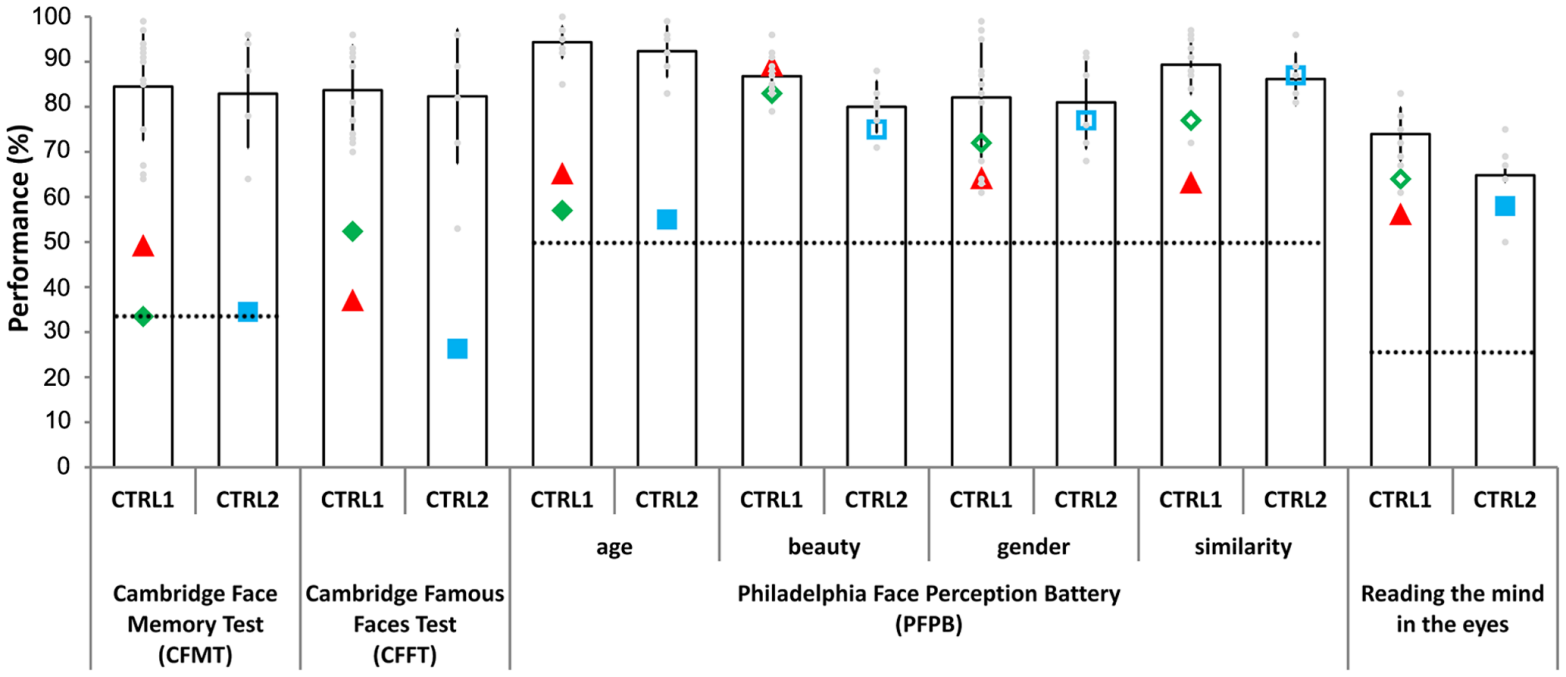
The results of neuropsychological assessment in the CP (congenital prosopagnosic) and neurotypical participants. Larger and filled symbols mark significant differences of the CP participants from the appropriate neurotypical groups. Error bars represent Standard Deviations, the dotted line represents the chance levels, if possible. CTRL1 - neurotypicals age-matched to CPd (triangles) and CPs (diamonds); CTRL2 - neurotypicals age-matched to CPf (squares). Light-gray circles represent the individual data of neurotypicals.

The results of the neuropsychological assessments are summarised in Fig. 1 and Table 1. To test the specificity of the impairments of the CP participants, we used the Doors task of the Doors and People test [63], which is sensitive to impairments of object recognition. On this test, each CP participant had normal performance (t-tests comparing CP participants against age-matched neurotypicals (older adults: n = 13, 6 male, mean age: 53.23±2.86; younger adults: n = 22, mean age: 22.91±2.6; (Racsmany et al., unpublished data)): CPf: *t*(12) = 0.58, *p* = 0.57; CPs: *t*(21) = −1.35, *p* = 0.19; CPd: *t*(21) = −0.24, *p* = 0.81), supporting the face specificity of their impairments.

**Table 1:** Characteristics (sex, age) of CP (congenital prosopagnosic) and neurotypical (CTRL1 and CTRL2) subjects as well as the results of the IQ (RSPM; Raven's Standard Progressive Matrices), the Cambridge Famous Faces Test (CFFT), the Cambridge face memory test (CFMT; z-scores [see 87]), the Philadelphia Face Perception Battery (PFPB) and the subjects' performance on Reading the Mind in the Eyes tests.

			CFFT			PFPB				
Subjects	Age	RSPM	familiar with	correctly identified	CFMT	age	beauty	gender	similarity	Reading the Mind in the Eyes
CPf, M	56	48	63%	**26%**	**−3.22**	**55%**	75%	77%	87%	58%
CPs, M	25	54	70%	**52%**	**−3.72**	**57%**	83%	72%	77%	64%
CPd, F	22	49	63%	**37%**	**−2.34**	**65%**	89%	64%	**63%**	**56%**
CTRL1/*N = 14/*, 7 M	24.5 (2.1)	52.8 (4.2)	81.7% (10.3)	83.6% (9.99)	0.63 (1.0)	94.4% (3.6)	86.8% (4.3)	82.1% (13.3)	89.4% (6.6)	73.9% (6.2)
CTRL2/*N = 6*/, 3 M	57,3 (5.4)	48.8 (3.6)	67,7% (17.2)	81.4% (16.64)	0.93 (1.0)	92.3% (5.7)	80.0% (5.7)	81.0% (0.3)	86.2% (5.8)	64.8% (8.3)

Bold denotes significant differences from the matched CTRL. Numbers in brackets refer to the standard deviations regarding CTRL1 and CTRL2.

The Raven Standard Progressive Matrices [86] showed that IQ is in the normal range for the CP participants (CPf: *t*(222) = −0.69, *p* = 0.49; CPs: *t*(222) = 0.39, *p* = 0.69; CPd: *t*(222) = −0.51, *p* = 0.61), not being different either from the Hungarian normal standard, or from the mean of the age and grade-matched groups CTRL1 and CTRL2 (CPf: *t*(5) = −0.21, *p* = 0.84; CPs: *t*(13) = 0.28, *p* = 0.79; CPd: *t*(13) = −0.87, *p* = 0.4). This suggests that their lower face recognition performance cannot be attributed to a more general intellectual impairment.

Face recognition performance of the CP participants on the Cambridge Famous Faces Test [66] was significantly worse than that of the neurotypicals (CPf: *t*(5) = −3.08, *p*<0.05; CPs: *t*(13) = −3.06, *p*<0.01; CPd: *t*(13) = −4.51, *p*<0.01). Similarly, the CFMT [65] revealed a significantly worse face memory of CP participants when compared to neurotypicals (CPf: *t*(5) = −3.77, *p*<0.01; CPs: *t*(13) = −4.16, *p*<0.01; CPd: *t*(13) = −2.87, *p*<0.01). While the scores on the CFMT of three CP participants were lower than the age-corrected values reported by Bowles and colleagues [87], the scores of the CTRL subjects were in the range of the age-corrected neurotypical subjects. The Philadelphia Face Perception Battery (PFPB) [64] revealed a heterogenic profile impairments in CP participants, however. While each family member showed a significantly lower performance in the “age” subtest when compared to neurotypicals (CPf: *t*(5) = −6.04, *p*<0.01; CPs: *t*(13) = −10.14, *p*<0.01; CPd: *t*(13) = −7.97, *p*<0.01), in other subtests, only CPd showed a significant impairment for similarity (“beauty”: CPf: *t*(5) = −0.81, *p* = 0.46; CPs: *t*(13) = −0.58, *p* = 0.57; CPd: *t*(13) = 0.34, *p* = 0.74, “similarity”: CPf: *t*(5) = 0.13, *p* = 0.9; CPs: *t*(13) = −1.81, *p* = 0.09; CPd: *t*(13) = −3.86, *p* = 0.01, “gender”: CPf: *t*(5) = −0.36, *p* = 0.73; CPs: *t*(13) = −0.73, *p* = 0.48; CPd: *t*(13) = −1.31, *p* = 0.21). Finally, the “Reading the mind in the Eyes” (Eyes Test [68]) test revealed a significantly lower performance for CPd but not for CPf and CPs (CPf: *t*(5) = −0.74, *p* = 0.49; CPs: *t*(13) = −1.54, *p* = 0.143; CPd: *t*(13) = −2.79, *p*<0.01). Overall, these results suggest that the CP participants all had an impaired recognition and memory for faces with various capacities of face perception within the normal range.

### 3.2. ERP results

Both CP and CTRL participants performed the category discrimination task during the ERP recordings well (88–95%). The average performance for CP participants was not different from that of the appropriate CTRL groups (CPf: *t*(5) = 0.18, *p* = 0.86; CPs: *t*(13) = −1.19, *p* = 0.25; CPd: *t*(13) = 1.27, *p* = 0.23).

#### 3.2.1. N170 latency

As expected [49], [84], [88], we found a significantly shorter N170 latency for FACE than for NOISE images for both the young and old neurotypical groups (main effect of Stimulus: (*F*(1,13) = 35.13, *p*<0.05, η^2^ = 0.73 and *F*(1,5) = 31.03, *p*<0.01, η^2^ = 0.861 for CTRL1 and CTRL2, respectively). This stimulus-specific change of the N170 latency, however, was significantly reduced in CPd over the LH and in CPf over the RH when compared to neurotypicals (Table 2).

**Table 2:** N170 ERP FND (face-noise difference) for left and right hemispheres (LH and RH).

		Amplitude		Latency	
		LH	RH	LH	RH
CPd	t(13)	**−3.514**	**−5.202**	**−6.547**	0.31
	p, p^b^	**0.004, 0.001**	**0.000, 0.001**	**0.000, 0.001**	0.761, 0.765
CPs	t(13)	1.267	**−5.132**	0.028	0.551
	p, p^b^	0.227, 0.247	**0.000, 0.01**	0.978, 0.982	0.591, 0.595
CPf	t(5)	**−5.611**	−2.469	0.674	**−3.699**
	p, p^b^	**0.002, 0.018**	0.057, 0.063	0.53, 0.56	**0.014, 0.017**

Second number (p^b^) shows the bootstrap p value. Bold numbers denote if One-samples T-test and bootstrap statistic (p^b^) show significant (*p*<0.05) differences between CP (congenital prosopagnosic) subject and matched neurotypicals.

#### 3.2.2. N170 amplitude

**Figure 2:**
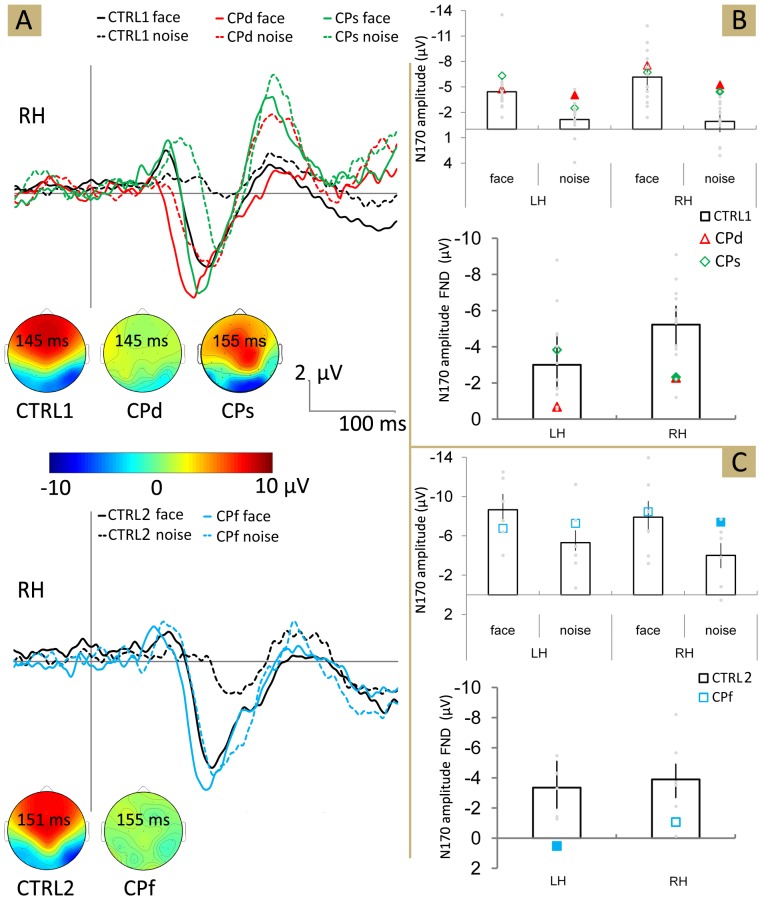
Grand-averaged ERPs. (A) ERPs evoked by faces (solid line) and their phase-randomised versions (noise stimuli; dashed line) at the right occipito-temporal electrode P10 separately for the younger (top) and older (bottom) participants. Black lines: CTRL1 and CTRL2; red: CPd, green: CPs, blue: CPf. Inserts show the voltage-maps of N170. (B) The N170 amplitude (top) and its FND (face-noise difference) for CTRL1, CPs and CPd as well as for CTRL2 and CPf. (C). Larger and filled symbols mark significant differences from the values of the matched neurotypicals. Error bars denote the 95% bootstrap confidence interval. LH - left hemisphere, RH - right hemisphere. Triangles, diamonds and squares symbolise the data of CPd, CPs and CPf, respectively, while light-gray circles represent the individual data of neurotypicals.

The N170 amplitude (Fig. 2) showed a significant main effect of Stimulus category for the young, as well as for the older neurotypicals (main effect of Stimulus: *F*(1,13) = 59.86, *p*<0.01, η^2^ = 0.82 and *F*(1,5) = 20.45, *p*<0.01, η^2^ = 0.95, for CTRL1 and CTRL2, respectively) due to significantly reduced N170 for the NOISE when compared to FACE, a result similar to previous findings [48], [49].

The stimulus-specific modulation of N170 amplitude was, however, reduced to a large extent in the CP participants with the exception of CPs over the left hemisphere, where it was not significantly different from the neurotypicals (Table 2, Fig. 2). Interestingly, the lower FND of CP participants was not due to the lower N170 amplitudes for FACE: face stimuli evoked similar N170 amplitudes in CPs (LH: *t*(13) = 2.501, *p* = 0.167; RH: *t*(13) = 0.713, *p* = 0.488), CPd (LH: *t*(13) = 0.42, *p* = 0.67; RH: *t*(13) = 1.69, *p* = 0.114) and CPf (LH: *t*(5) = 2.55, *p* = 0.074; RH: *t*(13) = 0.33, *p* = 0.72), when compared to the appropriate neurotypical groups. NOISE, on the other hand led to significantly higher N170 amplitudes for the RH of CPs (LH: *t*(13) = 1.96, *p* = 0.072; RH: *t*(13) = 5.278, *p*<0.001), bilaterally for CPd (LH: *t*(13) = 4.79, *p*<0.05; RH: *t*(13) = 6.55, *p*<0.01) and for CPf (LH: *t*(5) = 1.32, *p* = 0.264; RH: *t*(5) = 2.554, *p* = 0.074) when compared to neurotypicals. Thus it seems that the reduced face sensitivity of the N170 in CP participants originates from the enhanced N170 amplitude of NOISE condition. In order to understand the reason of this enhanced response we performed a single-trial analysis within the time-window, corresponding to the N170.

### 3.3. Intertrial variance of N170

**Figure 3:**
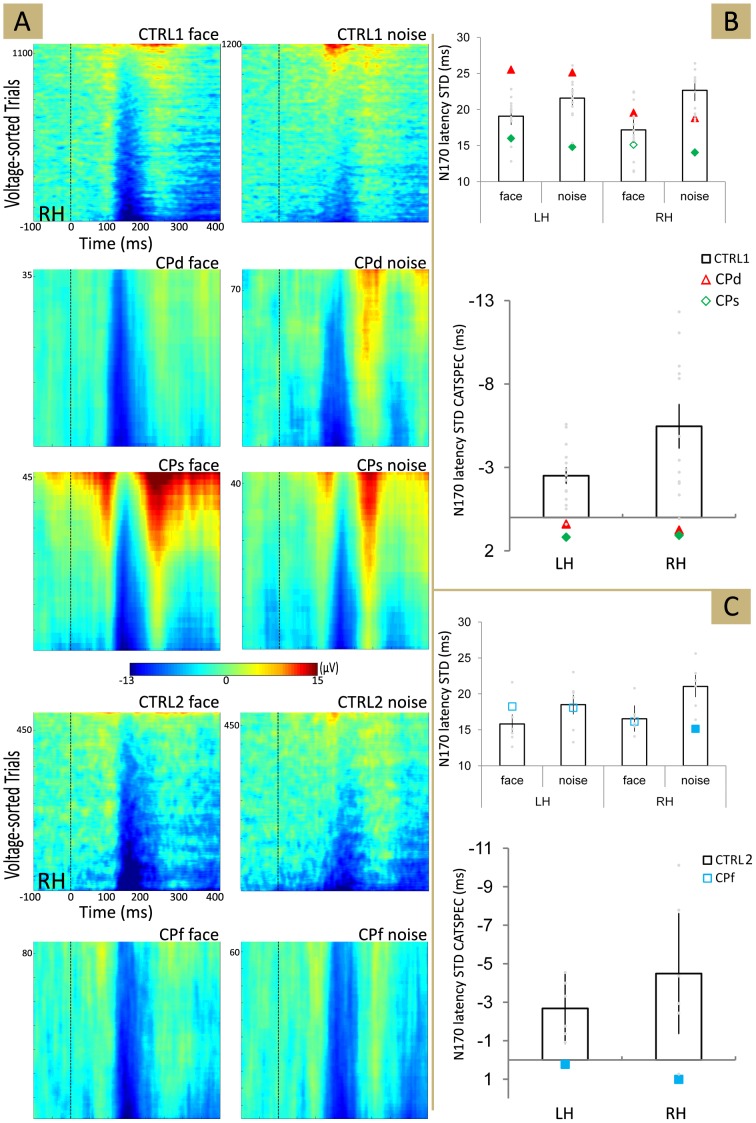
Trial-by-trial ERPs to illustrate the single-trial latency jitter differences. (A) Single-trial latency jitter differences for CTRL1, CTRL2 and the three CP (congenital prosopagnosic) participants, as well as for the face and noise stimuli separately. (B) and (C) Top: Columns mark the average (±95% bootstrap confidence interval) of the STD (standard deviation) of the N170 latency across CTRL1 (B) and CTRL (C) participants. Individual symbols represent the STD of the N170 latency for CPd (red), CPs (green) and CPf (blue) separately. Bottom: FND (face-noise difference) of trial-by-trial N170 latency, estimated as the difference of STD for face and noise stimuli (see Methods). Columns: the average FND of latency STD for CTRL1 (top) and CTRL2 (bottom). Larger and filled symbols mark significant differences from the values of the appropriate neurotypical groups. LH - left hemisphere, RH - right hemisphere.

Theoretically the stimulus category dependency of the N170 can either be due to the different amplitudes of single-trial responses or to the different stimulus onset-time locking of responses, expressed in the variance (or jitter) of its latency [31], [77], [89]. We found that the STD of the N170 latencies is significantly larger for NOISE when compared to FACE in both neurotypical groups (Fig. 3; main effect of Stimulus: *F*(1,13) = 34.68, *p*<0.01, η^2^ = 0.73 and *F*(1,5) = 23.44, *p*<0.01, η^2^ = 0.82 for CTRL1 and CTRL2, respectively) and for both hemispheres (main effect of Hemisphere; *F*(1,13) = 3.71, *p = *0.076, η^2^ = 0.22 and *F*(1,5) = 1.49, *p = *0.28, η^2^ = 0.23; Stimulus × Hemisphere interaction: *F*(1,13) = 0.03, *p* = 0.86, η^2^ = 0.01 and *F*(1,5) = 0.71, *p* = 0.44, η^2^ = 0.12 for CTRL1 and CTRL2, respectively). Prior studies suggest that the larger jitter of the latency leads to lower ERP component amplitudes [31], [77], [89], a conclusion supported by the significant negative correlation between intertrial latency jitter and average amplitude of the N170 (FACE: *r* = 0.62, *r* = 0.47 for the left and right hemispheres, respectively; NOISE: *r* = 0.52, *r* = 0.61 for the left and right hemispheres, respectively; *p*<0.05 for each correlation). In other words the larger the intertrial variance of the N170 latency, the smaller the amplitude of the averaged ERP component for the neurotypical participants. This suggests that the lower N170 amplitude for non-face stimuli is due, at least partially, to the less consistent latency from trial to trial [31], [77], [89].

As for CP participants the STD of the N170 latencies showed exactly the opposite pattern (Fig. 3): we found higher STD of latencies for FACE when compared to NOISE. The difference between CP and neurotypical participants was significant over both hemispheres (one-sample bootstrap comparison; see Table 3.) This suggests that in CP participants NOISE leads to more synchronous neural activity than FACE and than in neurotypicals and this might explain their higher N170 amplitudes and lower FND of N170 when compared to neurotypicals.

**Table 3:** ERP N170 FND (face-noise difference) for left and right hemispheres for mean and standard deviation of amplitudes and latencies of single trials.

		Latency				Amplitude			
		Mean		STD		Mean		STD	
		LH	RH	LH	RH	LH	RH	LH	RH
CPd	t(13)	**−2.364**	**3.485**	**−5.974**	**−4.612**	**−2.872**	**−4.251**	1.996	−0.058
	p, p^b^	**0.034, 0.057**	**0.004, 0.005**	**0.000, 0.002**	**0.000, 0.002**	**0.013, 0.014**	**0.001, 0.005**	0.067, 0.092	0.954, 0.955
CPs	t(13)	**3.108**	**8.599**	**−7.603**	**−4.849**	−0.8	**−6.788**	**−8.5**	**−17.954**
	p, p^b^	**0.008, 0.015**	**0.000, 0.001**	**0.000, 0.001**	**0.000, 0.002**	0.438, 0.458	**0.000, 0.001**	**0.000, 0.002**	**0.000, 0.001**
CPf	t(5)	−0.158	1.757	**−3.095**	**−3.444**	**−4.459**	**−2.053**	**−5.824**	−1.656
	p, p^b^	0.88, 0.888	0.139, 0.133	**0.027, 0.068**	**0.018, 0.038**	**0.007, 0.053**	**0.095, 0.122**	**0.002, 0.005**	0.159, 0.162

Second number (p^b^) shows the bootstrap p value. Bold numbers denote if One-samples T-test and bootstrap statistic (p^b^) show significant (*p*<0.05) differences between CP (congenital prosopagnosic) subject and matched neurotypicals.

### 3.4. Results of ITC and power analysis

#### 3.4.1. ITC

Intertrial synchrony (denoted as phase-locking factor as well [90]) is thought to reflect stimulus-related phase-locking of oscillations. It is related to the stimulus evoked ERP components and it increases if the phase of oscillations is synchronized to stimulus onset [51], [91], [92]. To test if the altered FND of single-trial latency variance of the evoked responses in CP participants comes together with an altered phase-locking as well we performed an ITC analysis. Since the effects are very similar for the theta (4–7 Hz) and alpha (8–12 Hz) bands (a result supported by previous studies, e.g. [93]) here we only present in detail the analysis of the theta-band.

**Figure 4:**
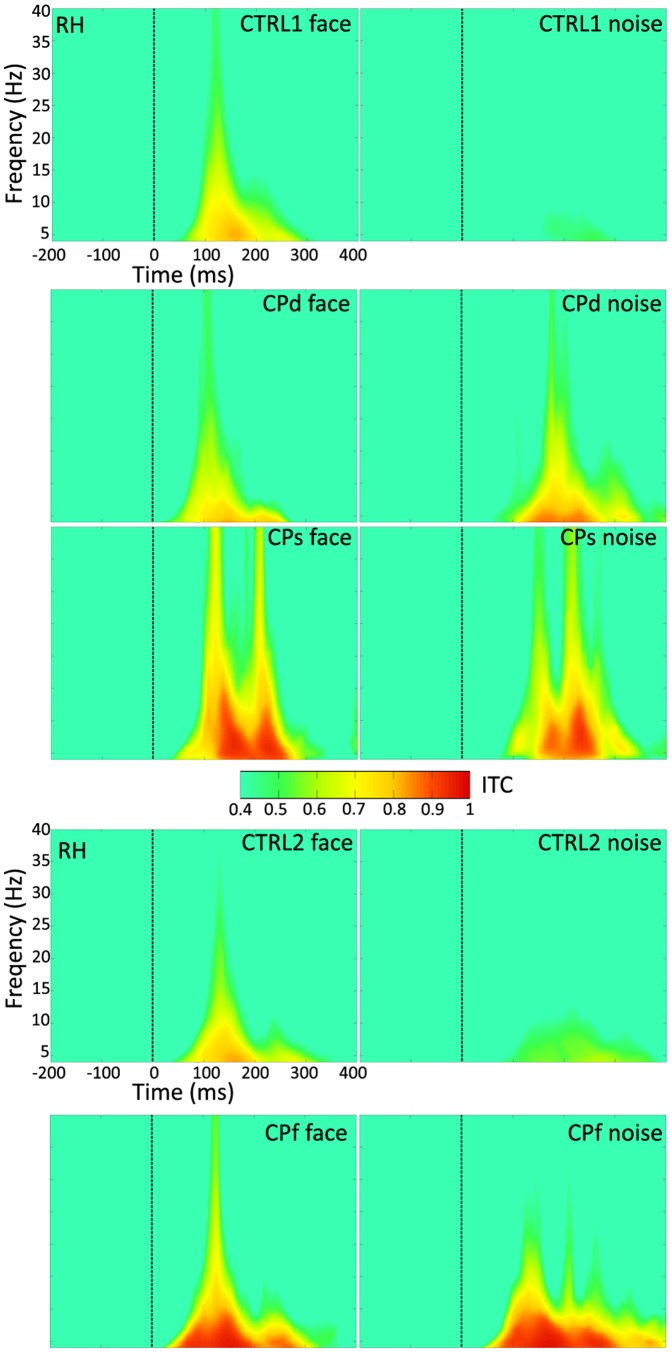
Time-frequency plot of the grand average ITC (intertrial coherence) for CTRL1, CTRL2 and for the three CP (congenital prosopagnosic) participants, as well as for face and noise stimuli separately. LH - left hemisphere, RH - right hemisphere.

**Figure 5:**
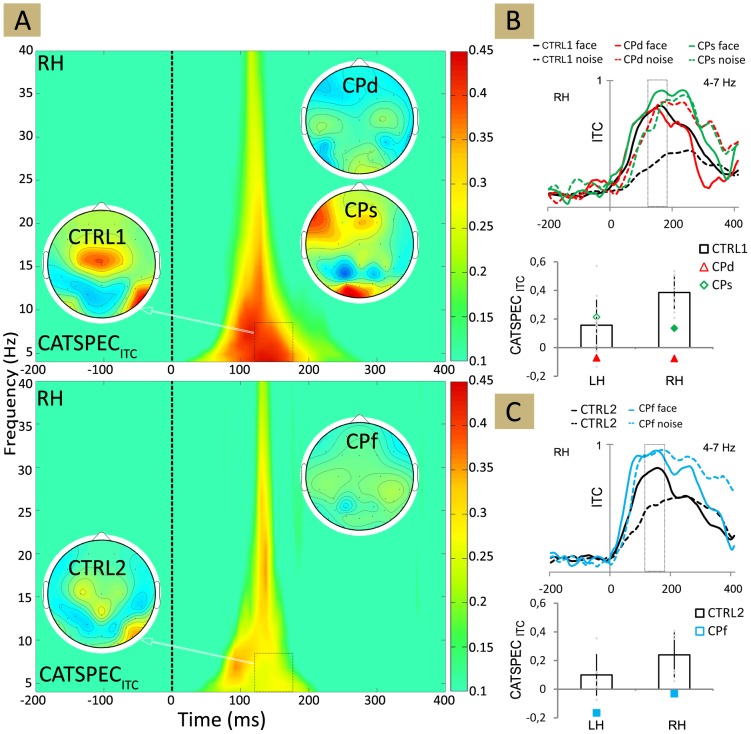
Time-frequency plot. (A) The plot showing the difference in ITC (intertrial coherence) for face minus non-face stimuli for CTRL1 (upper panel) and for CTRL2 (lower panel). Insets show the ITC topographies averaged over 130–190 ms and 4–7 Hz as indicated by the black dashed boxes. (B)-(C) Top: Mean theta-band ITC for face and noise stimuli. Bottom: The average difference in theta-band ITC for face minus non-face stimuli, calculated in the 130–190 ms time window for CTRL1 (B) and for CTRL2 (C). Black: CTRL1 and CTRL2; red: CPd, green: CPs, blue: CPf. Larger and filled symbols in the diagrams mark significant differences from the values of the matched neurotypicals. Error bars denote the 95% bootstrap confidence interval. LH - left hemisphere, RH - right hemisphere.

In agreement with the results of the N170 amplitude analysis we found that for the neurotypical participants the ITC was higher for FACE when compared to NOISE in the theta-band (Fig. 4; main effect of Stimulus: *F*(1,13) = 104.87, *p*<0.01, η^2^ = 0.89 and *F*(1,5) = 18.23, *p*<0.01, η^2^ = 0.78 for CTRL1 and CTRL2, respectively). Fig. 5 depicts the differential FND_ITC_ measure of the RH (see Methods), as well as its cortical distribution for CTRL1, CTRL2 and CP participants. The significantly larger ITC in FACE when compared to NOISE suggests that the responses are not only more aligned in latency but the phase of the theta-oscillations is also more constant at the onset of the FACE when compared to NOISE stimuli in the neurotypical participants. In addition, while the ITC was generally higher over the right when compared to the left hemisphere in the younger neurotypicals (main effect of Hemisphere: *F*(1,13) = 7.31, *p*<0.05, η^2^ = 0.36) it was similar for the two hemispheres in the older, CTRL2 group (main effect of Hemisphere: *F*(1,5) = 0.33, *p* = 0.59, η^2^ = 0.06). Interestingly, the FND_ITC_ was also more pronounced over the right hemisphere for the young (Stimulus × Hemisphere interaction: *F*(1,13) = 14.74, *p*<0.01, η^2^ = 0.53) but not for the older neurotypical groups (Stimulus × Hemisphere interaction: *F*(1,5) = 2.25, *p* = 0.19, η^2^ = 0.31).

As for the CP participants the results of ITC analysis support that of the single-trial latency analysis in the sense that the FND_ITC_ is significantly lower bilaterally for CPd and CPf and over the right hemisphere for CPs when compared to neurotypicals (Table 4; Fig. 5). This lack of face sensitivity of the ITC in CP participants, similarly to the one observed for the N170 amplitude came from the enlarged ITC for NOISE (Fig. 4). Face stimuli evoked similar or larger ITC in CPs (LH: *t*(13) = −0.75, *p* = 0.47; RH: *t*(13) = −3.17, *p*<0.05), CPd (LH: *t*(13) = 1.8, *p* = 0.11; RH: *t*(13) = 1.16, *p* = 0.27) and CPf (LH: *t*(5) = 1.17, *p* = 0.45; RH: *t*(5) = −2.67, *p* = 0.12), when compared to neurotypicals (Fig. 5a). NOISE, on the other hand led to significantly larger ITC values in the RH of CPs and CPf (CPs: *t*(13) = −9.02, *p*<0.01; CPf: *t*(5) = −4.54, *p*<0.01) and bilaterally in CPd (LH: *t*(13) = −2.14, *p*<0.05; RH: *t*(13) = −9.91, *p*<0.01) when compared to neurotypicals (Fig. 5b). Similarly to the N170 amplitudes, the ITC of the NOISE condition was also as large in CP participants as FACE evoked ITC of the neurotypicals (RH of CPs: *t*(13) = 0.22, *p* = 0.83; CPd: LH: *t*(13) = 0.66, *p* = 0.511; RH: *t*(13) = −0.72, *p* = 0.52; CPf: LH: *t*(5) = −1.14, *p* = 0.44; RH: *t*(5) = −3.31, *p* = 0.11). Thus it seems that the reduced FND_ITC_ of CP participants is related to the enhanced theta-band ITC of the NOISE condition.

**Table 4:** FND (face-noise difference) for LH and RH (left and right hemisphere) in terms of ITC (intertrial coherence) and ERSP (event-related spectral perturbation).

		ITC				ERSP			
		Theta		Alpha		Theta		Alpha	
		LH	RH	LH	RH	LH	RH	LH	RH
CPd	t(13)	**4.799**	**15.116**	**4.033**	**8.592**	**4.622**	**10.096**	1.84	**5.621**
	p, p^b^	**0.000, 0.001**	**0.001, 0.001**	**0.001, 0.005**	**0.000, 0.004**	**0.000, 0.003**	**0.000, 0.001**	0.089, 0.104	**0.000, 0.002**
CPs	t(13)	−1.205	**8.194**	−0.786	**3.886**	0.464	**2.269**	1.71	**2.542**
	p, p^b^	0.25, 0.248	**0.000, 0.001**	0.446, 0.447	**0.002, 0.01**	0.65, 0.62	**0.041, 0.039**	0.111, 0.122	**0.025, 0.044**
CPf	t(5)	**4.482**	**4.256**	2.376	**4.763**	1.96	**2.922**	−2.087	−0.019
	p, p^b^	**0.007, 0.019**	**0.008,** 0.081	0.063, 0.135	**0.005, 0.035**	0.107, 0.172	**0.033, 0.07**	0.091, 0.117	0.986, 0.986

Second number (p^b^) shows the bootstrap p value. Bold numbers denote if One-samples T-test and bootstrap (p^b^) statistic show significant (*p*<0.05) differences between CP (congenital prosopagnosic) subject and matched neurotypicals.

#### 3.4.2. Power

**Figure 6:**
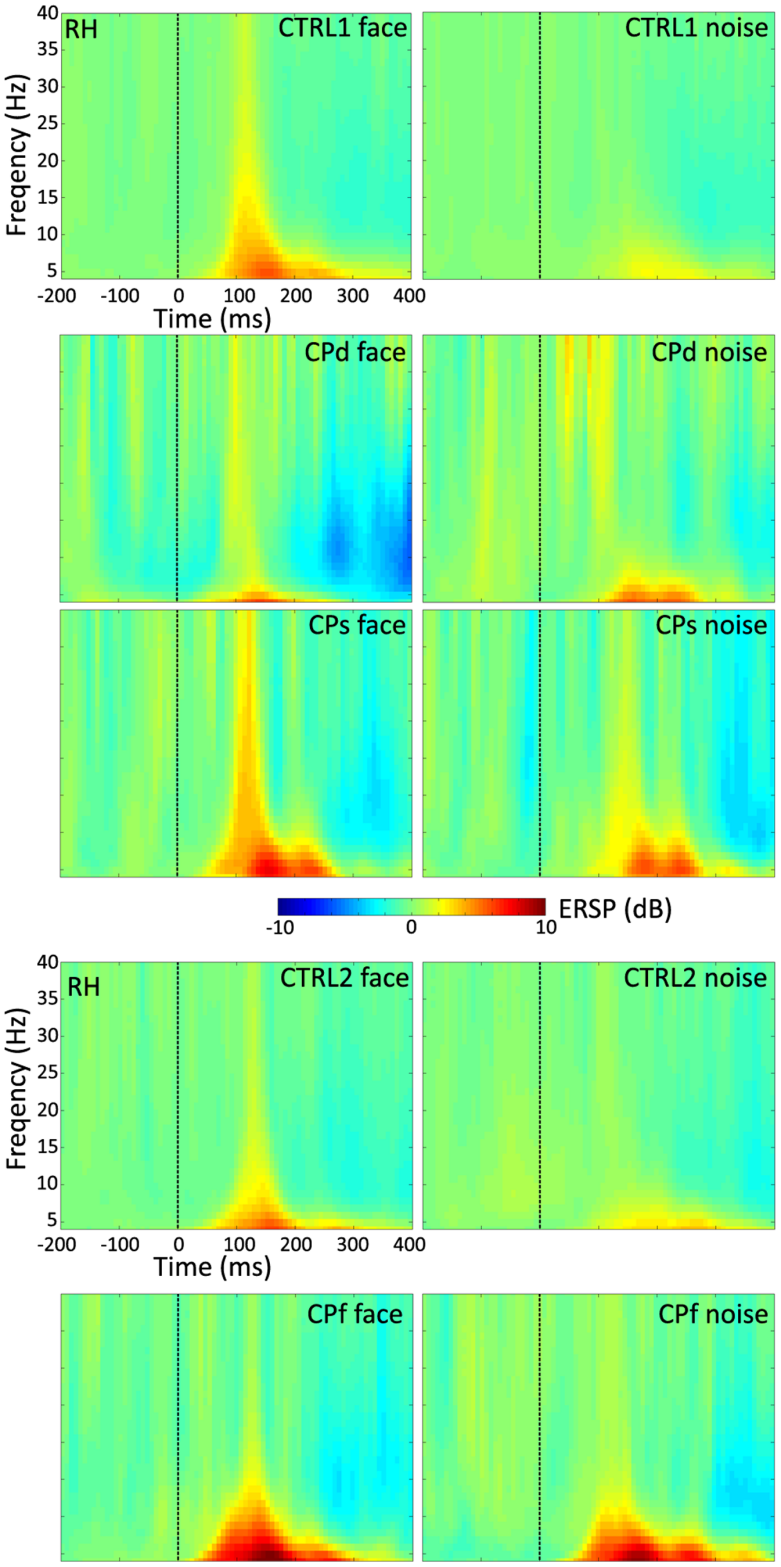
Time-frequency plot of the grand average event-related power for CTRL1, CTRL2 and for the three CP participants, as well as for face and noise stimuli separately. RH - right hemisphere.

**Figure 7:**
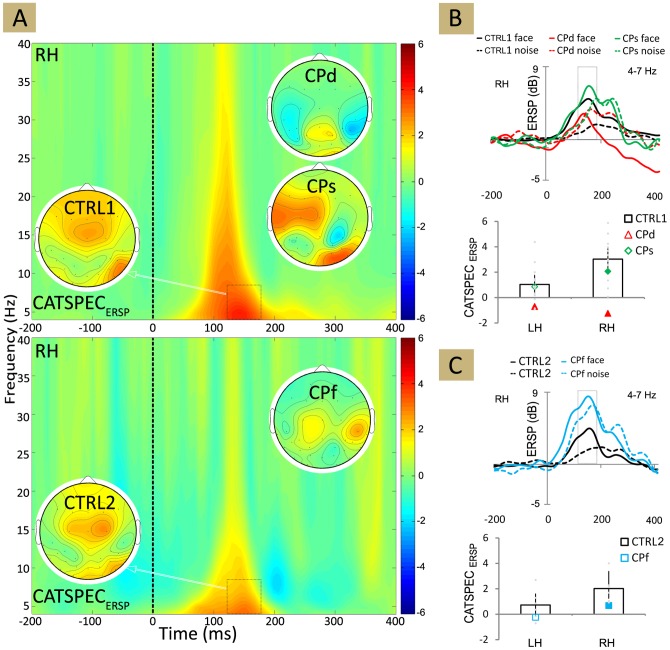
Time-frequency plot of ERSP. (A) The plot showing the difference in ERSP (event-related spectral perturbation) for face minus non-face stimuli for CTRL1 (upper panel) and for CTRL2 (lower panel). Insets show the ERSP topographies averaged over 130–190 ms and 4–7 Hz as indicated by the black dashed boxes. (B)–(C) Top: Mean theta-band ERSP for face and noise stimuli. Bottom: The average difference in theta-band ERSP for face minus non-face stimuli, calculated in the 130–190 ms time window for CTRL1 (B) and for CTRL2 (C). Black: CTRL1 and CTRL2; red: CPd, green: CPs, blue: CPf. Larger and filled symbols in the diagrams mark significant differences from the values of the matched neurotypicals. Error bars denote the 95% bootstrap confidence interval. LH - left hemisphere, RH - right hemisphere.

Power, expressed in the form of ERSP was significantly higher for FACE when compared to NOISE in both neurotypical groups as well (Fig. 6; main effect of Stimulus: *F*(1,13) = 43.02, *p*<0.01, η^2^ = 0.77; *F*(1,5) = 12.5, *p*<0.05, η^2^ = 0.71 for CTRL1 and CTRL2, respectively) and it was higher over the right when compared to the left hemisphere for the younger CTRL1 group (main effect of Hemisphere: *F*(1,13) = 24.64, *p*<0.001, η^2^ = 0.65). Fig. 7 depicts the differential FND_ERSP_ of the RH (see Methods), as well as its cortical distribution for CTRL1, CTRL2 and CP participants. The significantly larger ERSP in FACE when compared to NOISE supports the results of the N170 amplitude analysis in the neurotypical participants.

The FND_ERSP_ was significantly lower bilaterally for CPd and over the right hemisphere for CPs and CPf when compared to neurotypicals (Table 4; Fig. 6). This lack of face sensitivity of the ERSP in CP participants, similarly to what we have observed for the N170 amplitude, also came from the larger ERSP for NOISE (Fig. 6), suggesting that the reduced FND_ERSP_ of CP participants relates to the enhanced theta-band power of the NOISE condition.

## Discussion

Our behavioural results support previous findings that suggested the familial aggregation of impaired face perception (see e.g. [21], [24], [41]–[43]). In addition, in the present study, we provide further electrophysiological evidence indicating that face perception impairments can be found in more than one member of the same family, stretching across generations, supporting the role of the hereditary factors in developmental prosopagnosia. Face recognition performance is strongly impaired in three members of the same family when compared to age-matched neurotypicals. Specifically, we found severe impairment in the recognition of famous faces in each CP participant, similarly to previous studies of CP and AP ([12], [24], [94], [95], for a review see [96]). In addition, and in line with recent results [14], [42], [97] we found a heterogeneous perceptual profile across the three CP participants. Although previously impaired gender judgments has been reported (e.g. [98]), the Philadelphia Face Perception Battery [64] revealed a significant impairment in all CP participants only in the age subtest, with unimpaired beauty, gender or similarity judgements.

While most individuals with prosopagnosia do not exhibit difficulties in processing facial emotions ([40], [41], [99], [100], for a review see [101]), we identified a degree of impaired emotion processing for CPd, using the Eyes Test [68]. Recent studies (e.g. [102]) showed that identity and emotion recognition are both accompanied by an enhanced activity of the Fusiform Face Area as well (for a review of unitary face perception models see [103]), but traditional face perception models assume different neural pathways of identity and emotional face processing [45], [46], [104]. In line with the theoretical implications of classic models, the decreased facial identity and emotion recognition performance may be caused by the disruption of early stages of face processing in these CP participants. Altogether, the neuropsychological tests suggest that, in addition to their impaired face recognition capacities, CP participants show a somewhat heterogeneous profile of face perception capacities, even within the same family. However, in spite of this behavioural heterogeneity, the electrophysiological findings of the present study as well as the functional properties of their core face processing system [44] are surprisingly similar across family members with CP, suggesting a shared heritable neural basis.

To test the electrophysiological correlates of the face recognition impairments of CP participants we tested if their N170 ERP component shows typical face sensitivity (i.e. larger amplitude for faces compared to non-face stimuli) commonly observed in neurotypical participants. Our results show similar N170 amplitudes for faces and random noise stimuli, suggesting attenuated sensitivity to faces. This is in line with previous studies demonstrating impaired face selectivity of the N/M170 in AP [82], [105], DP ([34], but see [35]–[38]) and CP participants [13], [39], [40]. Interestingly, the reduction of the face sensitivity of N/M170 in CP participants is caused by increased amplitude for noise, rather than by reduced amplitude for faces. This result is in line with previous studies of N/M170 in prosopagnosics. With the exception of a single case (JS [37]), the lack of face selectivity of the N/M170 was caused by an enhanced neural activity for the non-face stimuli rather than by a decreased signal to faces in all of the above mentioned prosopagnosic participants. It is worth mentioning that for one CP participant (MZ [39]) the originally face-like ERP for non-face objects decreased after an extensive face-perceptual training, while the face evoked N170 remained unchanged. Moreover, our findings in CP participants show a parallel to a recent study showing that (1) N170 FND is substantially reduced in normal participants, when face or non-face images are shown under high attentional load, and that (2) this reduction of N170 face sensitivity is, to a considerable extent, due to an increase in the N170 amplitude elicited by non-face stimuli [106]. Altogether these results suggest that the neural processes, reflected in N170 are less selective for faces in DP/CP than in neurotypicals. This suggests that impaired face recognition in the present CP participants is related to a deficit of early detection and structural encoding stages of face processing [56], [107] which are involved in efficient selective streaming of information into category-specific processing mechanisms (see [40], [107]).

What could be the reason behind the enhanced N170 observed for non-face stimuli in DP/CP? Theoretically, the amplitude of an ERP component can reflect (1) the increase in EEG amplitude, (2) the accuracy of phase resetting of ongoing EEG oscillations or (3) the consistency of the response latency across trials for the stimuli. Please note, that the relative contribution of these factors in creating an averaged ERP component is under heavy ongoing debate (for a review see [108]), and this issue is beyond the scope of the present study. As for the face sensitivity of the N170 in neurotypical participants current results suggest that a larger increase of EEG amplitude [51] with a more consistent latency [31] are more likely to contribute to the effect than a more precise intertrial phase-realignment. To our best knowledge so far no study examined the mechanisms of altered face sensitivity of N170 in individuals with prosopagnosia. We found that the response of the CP participants was larger in power, better aligned and more synchronised to the appearance of the noise images when compared to neurotypicals. While the latency jitter of the N170 was significantly larger for non-face stimuli when compared to faces in neurotypicals, it was smaller and similar for both categories in CP participants. Parallel to this, both the power and the ITC values were similarly high for the two stimulus categories in CP participants. Therefore our results suggest that the firing of face specific neurons is similarly high and synchronised to the onset of face and non-face stimuli, explaining, at least partially, the similar N170 amplitudes and the reduced face sensitivity of the CP participants. This argues strongly that early structural encoding processes, reflected in N170 [32], [109]–[112], fail to discriminate between face and non-face stimuli. Accordingly, our results suggest that the impairment seen in the present cases of congenital prosopagnosia results from an early filtering deficit in the sense that the face processing system is unable to differentiate between face and non-face stimuli [39], [40]. However, it is worth noting that this phenomenon is not general for every case of CP/DP (e.g. see most of the cases in the study of Towler et al. [35]), suggesting the heterogeneity of the neural background of the impairment. Whether this heterogeneity is due to the existence of different subtypes of developmental prosopagnosia will require further studies.

In summary, the present study provided novel insights into the neural mechanisms underlying the reduced face-sensitive processes, reflected in the N170 ERP component, in individuals with congenital prosopagnosia. Further, studies will be necessary to estimate the generality of this finding in other families showing prosopagnosia across several generations.
